# Identification of potential key genes and pathways associated with the Pashmina fiber initiation using RNA-Seq and integrated bioinformatics analysis

**DOI:** 10.1038/s41598-021-81471-6

**Published:** 2021-01-19

**Authors:** Basharat Bhat, Mifftha Yaseen, Ashutosh Singh, Syed Mudasir Ahmad, Nazir A. Ganai

**Affiliations:** 1 Division of Animal Biotechnology, Sher-e-Kashmir University of Agricultural Sciences and Technology of Kashmir, FV.Sc and A.H, Shuhama, Jammu and Kashmir 190016 India; 2 Department of Life Science, Shiv Nadar University, Gautam Buddha Nagar, UP 201314 India; 3 Department of Interdisciplinary Sciences, Jamia Hamdard University, Delhi, 110062 India

**Keywords:** Computational biology and bioinformatics, Bioinformatics

## Abstract

Pashmina goat (*Capra hircus*) is an economically important livestock species, which habitats the cold arid desert of the Ladakh region (India), and produces a princely animal fiber called Pashmina*.* The Pashmina goat has a double coat fleece as an adaptation to the very harsh cold winters the outer long coarse hair (guard hair) produced from primary hair follicles and the inner fine Pashmina fiber produced from secondary hair follicles. Pashmina fiber undergoes a circannual and synchronized growth cycle. In the present study, we analyzed transcriptome profiles from 10 different Pashmina goats during anagen and telogen to delineate genes and signaling pathways regulating active (anagen) and regressive (telogen) phases of the follicle growth. During anagen, 150 genes were expressed at significantly higher levels with log (FC) > 2 and p_adj_ < 0.05. The RNA seq results were subjected to qRT-PCR validation. Among the nine genes selected, the expression of *HAS1*,* TRIB2*, *P2RX1*. *PRG4*, *CNR2*, and *MMP25* were significantly higher (p < 0.05) in the anagen phase, whereas *MC4R*, *GIPC2*, and *CDO1* were significantly expressed (p < 0.05) in the telogen phase which supports and validates the gene expression pattern from the RNA-sequencing. Differentially expressed genes revealed that Pashmina fiber initiation is largely controlled by signaling pathways like Wnt, NF-Kappa, JAK-STAT, Hippo, MAPK, Calcium, and PI3K-Akt. Expression of genes from the Integrin family, Cell adhesion molecules, and ECM-receptors were observed to be at much higher levels during anagen. We identified key genes (*IL36RN*, *IGF2*, *ITGAV*, *ITGA5*, *ITCCR7*, *CXCL5*, *C3*, *CCL19*, and *CXCR3*) and a collagen cluster which might be tightly correlated with anagen-induction. The regulatory network suggests the potential role of *RUNX3, NR2F1/2,* and *GATA* family transcription factors in anagen-initiation and maintaining fiber quality in Pashmina goats.

## Introduction

Pashmina goat (*Capra hircus*) is an economically important animal genetic resource adapted to very harsh cold arid agro-climatic conditions of Ladakh region (Jammu and Kashmir—India). The cold desert of Ladakh has a very short growing season and remains landlocked for more than half of the year. The mercury level of this land-locked high-altitude habitat (5500–6000 m above mean sea level) of Pashmina goat fluctuates between + 35 °C (short summers) and – 40 °C (long winters). Under these stressful conditions (cold, arid, hypoxic and scanty vegetation), Pashmina goats remain active with different adaptation strategies. This goat produces the world’s finest (11–14 µ) natural fibre (Pashmina fiber) which is used in the making of world-famous Pashmina/Cashmere shawls.

Pashmina fibre is the soft under-coat of the Pashmina goat mixed with coarse outer coat known as guard hair^1^. The guard hair develops from primary hair follicles (PHF) and Pashmina from secondary hair follicles (SHF). Pashmina fibre provides softness and luster to the product and has the glamour of being very rare. The unique fiber properties like fineness, texture and warmness are co-related with Pashmina genetics and breeding traits. The Pashmina fibers shed annually and are harvested by combing.

Hair follicles (HF) undergo cycles of the rapid growth phase (anagen), regression phase (catagen) and no-growth phase (telogen) throughput the lifetime. Hair follicles produce entire new hair shaft during the anagen phase. However, the underlying mechanism remains classical in the area^2^. Different approaches and animal models have been utilized to decipher the genetic basis of fiber transition^3–9^. However, asynchronous hair growth, the difference in skin anatomy and physiology restricts the identification of key molecular determinants mediating fiber transition.

Regulation of the hair cycle involves complex signaling interactions between Wnt (Wingless Integrin), Shh (Sonic HedgeHog), Notch, BMP (Bone Morphogenic Protein) and other signaling Pathways^6^. WNT^10,11^ and Shh^12^ signaling is indispensably important for new anagen, whereas BMPs^13^ have been implicated in follicle differentiation. However, signaling pathways involved in hair follicle cycling are not sufficiently studied to date and new pathways are yet to be discovered for the proper understanding of the hair cycle^2^.

The secondary follicles of Pashmina goat present an excellent model for studying hair biology due to the circannual fiber cycle and synchronized fiber growth^14^. This follicle is also an excellent model for studying diverse cellular, molecular and biological processes^15,16^. In the present study transcriptomic profiling of skin biopsies containing secondary hair follicles were utilized to identify key genes and signaling pathways involved in hair follicle transition from no-growth phase (telogen) to growth phase (anagen).

## Materials and methods

### Experimental design

Changthangi goats were selected from the flock maintained at High Mountain Arid Agricultural Research Institute (HMAARI) Stakna Leh (Ladakh). All animals were kept under the identical conditions of natural photoperiod and natural temperatures. This study was approved by the Animal Welfare and Ethics Committee of the Sher-e-Kashmir University of Agricultural Science and Technology of Kashmir (SKUAST-K). All animal experiments were conducted in strict accordance with the rules and guidelines outlined by the SKUAST-K Animal Welfare Committee. As per the guidelines of the committee, the skin samples were collected aseptically using skin biopsy punch under local anaesthesia with minimal pain and discomfort to the animal.

Ten unrelated Pashmina goats of the same age (24 months) and sex (males) were repeatedly sampled during anagen (October active growth phase) and telogen (March—resting phase, before combing)^17^. The skin samples containing Pashmina follicles were collected from the flanking region of each goat. The skin biopsies samples were snap-frozen in liquid nitrogen and shipped to the laboratory in RNA-later for processing. All samples corresponding to a particular stage were collected at the same time.

### Total RNA extraction, library construction, and sequencing

Total RNA was isolated from the skin tissues using the Trizol method (Invitrogen, USA) according to the manufacturer’s protocol. RNA samples with a RIN value greater than 7.0 were selected for RNA-sequencing (RNA-seq). A total of 19 (10 anagen and 9 telogen) samples were further carried forward for the analysis as one of the samples from telogen did not qualify the minimum quality criteria for RNA-seq. For cDNA library preparation and sequencing, RNA samples were stored at − 80 °C. Approximately 4 µg of total RNA was used to prepare the RNA sequencing library using the TruSeq RNA Sample Prep Kits (Illumina) as per the kit’s protocol. Agilent-tape station plots were used at every step to assess mRNA quality, enrichment success, fragmentation sizes, and final library sizes. Finally, the amplified fragments were sequenced using Illumina HiSeq 2500 to obtain 2 × 100 bp paired-end (PE) reads.

### Data analysis

The raw reads were pre-processed to remove the adapter sequences, low-quality reads and low-quality bases filtration towards 3′- end using cutadapt program v2.10^18^. Filtered reads were mapped to *Capra hircus* reference assembly ARS1 using Hisat program v2.20 (release date 2/6/2020)^19^. Quality control and alignment statistics (Supplementary Table 1) suggest the sequencing data were uniform among all sets of samples. Differential expression analysis between two contrast groups was performed using edgeR v3.30.3^20^ using Trimmed Means of M-values (TMM) normalization method and paired test, after removing low expressed genes across all samples. DEGs between different cycling stages were screened based on the threshold of FDR corrected P-value < 0.05 and absolute log2 (fold change) > 1. Significantly, dysregulated genes were subjected to functional annotation and pathways enrichment analysis using KOBAS server v3.0^21,22^.

### Validation of DEGs with qPCR

cDNA was synthesized from 0.5 μg of the same total RNA used in RNA-sequencing using the Revert Aid First Strand cDNA Synthesis Kit (Thermo Scientific, USA) as per the manufacturer's protocol. qPCR reactions were run on a Roche Lightcycler 480 II in a 20-μl reaction containing 0.5 μl of cDNA template, 10 μl of 2 × SYBR Green Master Mix, 0.3 μl of each primer (10 μmol/μl) and 8.9 μl nuclease-free water. The amplification program consisted of one cycle at 95 °C for 10 s, followed by 40 cycles of 95 °C for 15 s and 55 °C for 34 s. The qPCR reactions for each gene were performed with three biological replicates. Relative gene expression was normalized to the expression of goat GAPDH and calculated with the 2^−ΔΔCT^ method^23^. The expression levels of the genes obtained in RNA-seq and qPCR were compared with Pearson correlation coefficient.

## Results

### Primary processing of reads and differentially expressed genes

**Figure 1:**
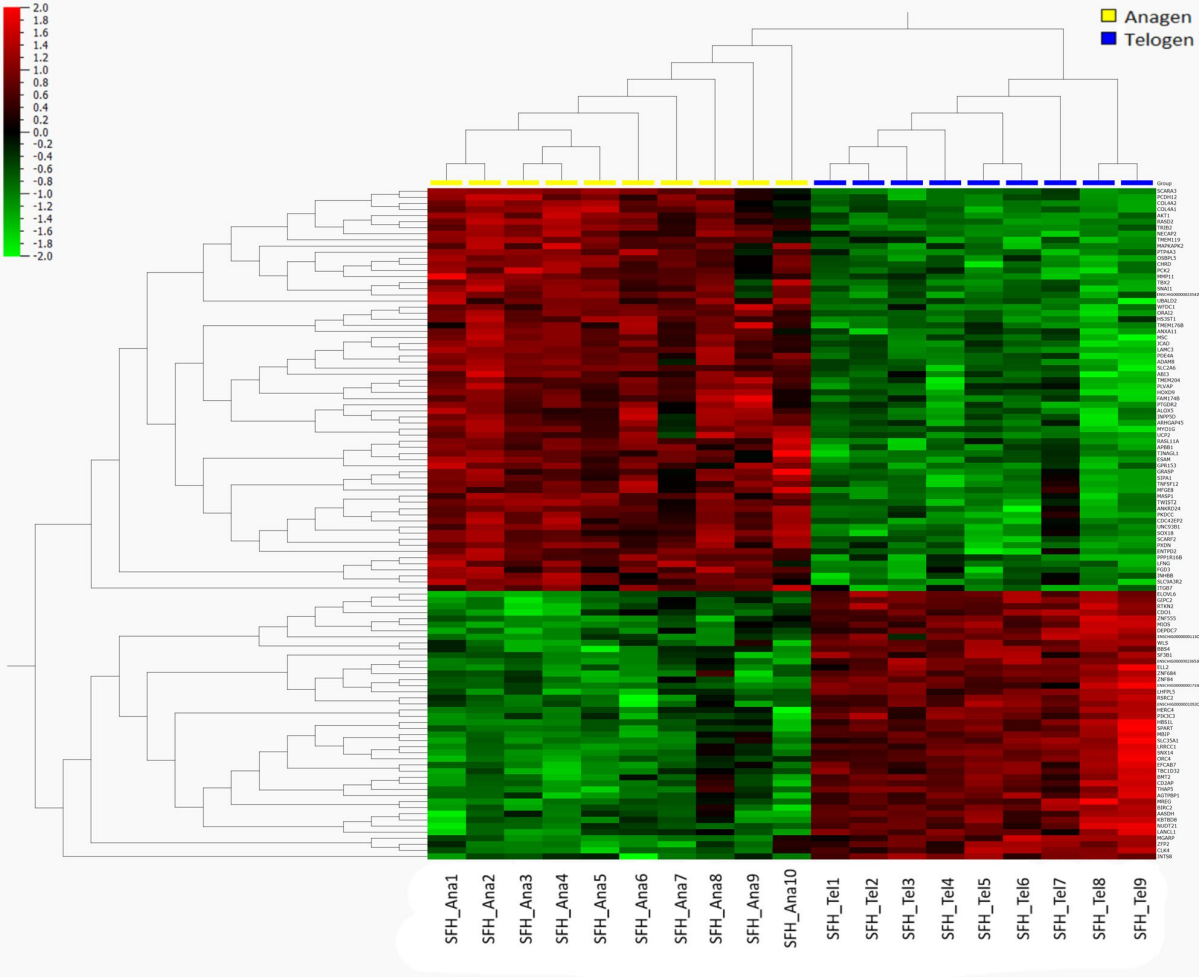
Hierarchical clustering and heatmap of top DEGs between two contrast groups. Columns indicate individual samples, rows represent each differentially expressed gene, and the color scale represents the relative expression level of the differentially expressed genes.

A summary of sequencing read alignments to the reference *Capra hircus* genome assembly ARS1 is presented in Supplementary Table 1. On average, 97% of the total reads were mapped successfully. Among the aligned reads, 98% were mapped to unique genomic regions. The characteristics of RNA-seq data were determined using PCA plots (Supplementary Fig. 1), showing a clear segregation and clustering of samples from different stages. Based on differential expression analysis using edgeR, 1094 genes were significantly dysregulated (log (FC) > 1, p_adj_ < 0.05). Of the DEGs, a total of 748 were significantly up-regulated and 346 were down-regulated in the anagen (Supplementary Table 2) as compared to telogen. Two-dimensional hierarchical clustering and heatmap of top differentially expressed genes (q-value) were shown in Fig. 1.

### Gene ontology and pathway enrichment analysis of DEGs

To gain insight into the biological processes and pathways that could mediate the fiber growth in Pashmina goats, Gene ontology (GO) and KEGG enrichment analysis were performed. The top enriched GO terms (Supplementary Table 3) for the DEGs are related to biological processes like ‘*immune system process’*, ‘*cell migration’*, ‘*developmental process’*, ‘*metabolic process’* and molecular function terms like ‘*protein binding’, ‘catalytic activity’, ‘growth factor binding’* and *‘transporter activity’* suggesting the potential role of growth factors and immune response genes for initiating and maintaining fiber activity. Anagen induction showed enrichment of 22 KEGG pathways (Table 1) which include signaling pathways like PI3K-Akt, NF-kappa B, MAPK, Hippo Wnt and JAK-STAT.

**Table 1:** Enriched KEGG pathways for DEGs between anagen and telogen.

Pathways	FDR corrected P-value	Percentage enrichment	Genes
Cytokine-cytokine receptor interaction	1.10E−11	14.24149	CCL2, TNFSF12, LIF, CD40LG, IL10RA, TNFSF14, IL1F10, IL36RN, CD4, CCR2, CCR4, CCR7, IL1A, TNFSF13B, CSF2RB, CCL5, CSF1R, CCR10, THPO, CXCR3, INHBB, OSM, CXCR6, CCL14, IFNE, IL36G, TNFSF4, TGFB1, IL5RA, ACVRL1, IL2RB, IL7R, CD40, LTB, LTA, FASLG, TNFRSF1B, EPOR, IL2RA, BMP5, CXCL5, ACKR4, CCL19, IL21R, CLCF1, TNFRSF6B
PI3K-Akt signaling pathway	3.24E−07	10.72386	PCK2, ITGA9, COL4A1, PDGFRB, CSF1R, LAMA2, ITGA5, ITGA11, LAMB2, FLT1, THBS4, THBS2, PIK3CG, PIK3R5, OSM, JAK3, FGF16, ANGPT4, IGF2, IGF1R, NOS3, ITGB7, IL7R, FASLG, COL4A2, TNC, LAMC3, KITLG, COL6A3, EPOR, IL2RA, IL2RB, FN1, ITGAV, F2R, EREG, CREB3L4, VWF, COL6A2, LPAR6
ECM-receptor interaction	7.52E−07	20.68966	COL4A1, ITGA9, LAMA2, COL6A2, FN1, ITGB7, THBS4, HSPG2, THBS2, ITGA5, ITGAV, ITGA11, COL4A2, TNC, LAMC3, VWF, LAMB2, COL6A3
Cell adhesion molecules (CAMs)	9.32E−07	15.09434	ITGA9, CDH15, CLDN15, NRCAM, CD34, CD4, CD226, CD2, L1CAM, CD40LG, CD86, ICAM1, ITGAL, CDH5, CDH4, CLDN7, ITGB2, ITGB7, CD40, CD8A, ITGAV, PTPRC, ITGAM, ESAM
Neuroactive ligand-receptor interaction	6.92E−06	9.917355	PTH1R, CCK, GABRR2, CHRNA10, C5AR1, ADCYAP1, P2RX1, UTS2R, GPR35, MC4R, ADORA2B, CNR2, ADRB1, TAC1, RXFP2, P2RY10, ADRA2A, PTGER3, GAL, HRH2, C3, VIPR2, GRM2, TBXA2R, GRID2, OXT, PMCH, F2RL3, S1PR4, APLNR, C3AR1, DRD5, NPB, F2R, HCRTR1, LPAR6
Focal adhesion	8.17E−06	12.62626	ITGA9, COL4A1, PDGFRB, LAMA2, ITGA11, LAMB2, FLT1, THBS4, THBS2, PARVB, PARVG, IGF1R, RAC2, ITGB7, SHC3, ITGA5, COL4A2, TNC, LAMC3, VWF, FN1, VAV1, ITGAV, COL6A3, COL6A2
NF-kappa B signaling pathway	1.86E−05	16.66667	CD40LG, TIRAP, CD14, CCL19, CARD11, TNFSF14, CD40, LTB, LTA, ZAP70, ICAM1, PRKCQ, RELB, LYN, LAT, BTK, TNFSF13B
Rap1 signaling pathway	3.10E−05	11.57407	RASGRP2, PDGFRB, APBB1IP, PLCB2, GNAI2, FLT1, ADORA2B, ARAP3, RAP1GAP, CSF1R, SIPA1, FGF16, ANGPT4, IGF1R, ITGB2, RAC2, LAT, LCP2, KITLG, ADCY4, VAV1, F2R, ITGAL, ITGAM, F2RL3
cAMP signaling pathway	0.000169188	10.52632	NFATC1, ADCYAP1, ATP2A3, ADCY10, RAC2, GNAI2, ARAP3, ADRB1, CNGB3, CAMK4, PTGER3, VIPR2, NPR1, LIPE, OXT, CNGA3, PDE10A, ADCY4, VAV1, DRD5, F2R, CREB3L4, PDE4A, CFTR
MAPK signaling pathway	0.00685971	7.876712	RASGRP2, NFATC1, RASGRP4, PDGFRB, TGFB1, IL1A, FLT1, CACNA1H, CSF1R, DUSP9, RELB, FGF16, ANGPT4, IGF2, IGF1R, RAC2, STK3, FASLG, KITLG, PTPN7, RPS6KA2, CD14, EREG
Calcium signaling pathway	0.006925474	8.866995	ORAI2, TBXA2R, ADORA2B, NOS3, F2R, PLCB2, ADCY4, DRD5, CAMK4, PDGFRB, PTGER3, ITPKA, ADRB1, GNA14, HRH2, CACNA1H, P2RX1, ATP2A3
Ras signaling pathway	0.0081218	8.264463	IGF2, RASGRP2, IGF1R, RAC2, RASGRP4, SHC3, PLA2G12B, CSF1R, FGF16, PLA2G2E, ZAP70, PLA2G2F, LAT, FASLG, KITLG, RASA3, PDGFRB, RASAL3, ANGPT4, FLT1
Protein digestion and absorption	0.01083711	10.61947	COL15A1, SLC9A3, KCNQ1, COL5A3, KCNN4, COL13A1, COL4A2, COL4A1, COL24A1, COL6A3, COL6A2, SLC1A1
Arachidonic acid metabolism	0.011034945	12.04819	PTGS1, TBXAS1, PLA2G12B, ALOX5, EPHX2, PTGIS, ALOX15, PLA2G2E, PLA2G2F, PTGES
Melanogenesis	0.014067417	10.78431	WNT2B, ADCY4, PLCB2, CREB3L4, WNT3, WNT2, WNT9A, FZD9, WNT5B, KITLG, GNAI2
Hippo signaling pathway	0.014200591	9.271523	WNT2B, TGFB1, BMP5, ITGB2, WNT3, WNT2, STK3, WNT9A, FZD9, DLG2, WNT5B, WTIP, SERPINE1, CTNNA3
cGMP-PKG signaling pathway	0.014896486	8.87574	ADCY4, NFATC1, PLCB2, NOS3, NFATC4, NPR1, ATP2A3, KCNJ8, ADRA2A, PIK3CG, ADRB1, PIK3R5, CREB3L4, PDE2A, GNAI2
Phospholipase D signaling pathway	0.014927328	9.210526	ADCY4, PDGFRB, FCER1G, FCER1A, SHC3, MS4A2, PLCB2, PIK3CG, F2R, PIK3R5, KITLG, LPAR6, CYTH4, GRM2
Wnt signaling pathway	0.021435141	8.695652	WNT2B, NFATC1, PLCB2, NFATC4, RSPO4, WNT3, WNT2, LGR5, SOX17, RSPO2, WNT9A, FZD9, WNT5B, RAC2
Jak-STAT signaling pathway	0.025691752	7.920792	IL7R, IL2RA, IL2RB, STAT4, SOCS1, IL10RA, PDGFRB, IFNE, IL21R, IL5RA, THPO, LIF, OSM, JAK3, CSF2RB, EPOR
Signaling pathways regulating pluripotency of stem cells	0.035422505	8.633094	WNT2B, LIF, PCGF6, WNT3, WNT2, IGF1R, INHBB, DUSP9, JAK3, WNT9A, FZD9, WNT5B
Purine metabolism	0.049889538	8.148148	ADCY4, PKLR, ENTPD2, NPR1, ADA2, AK5, PDE2A, PDE6A, ADCY10, PDE4A, PDE10A

### Network analysis

**Figure 2:**
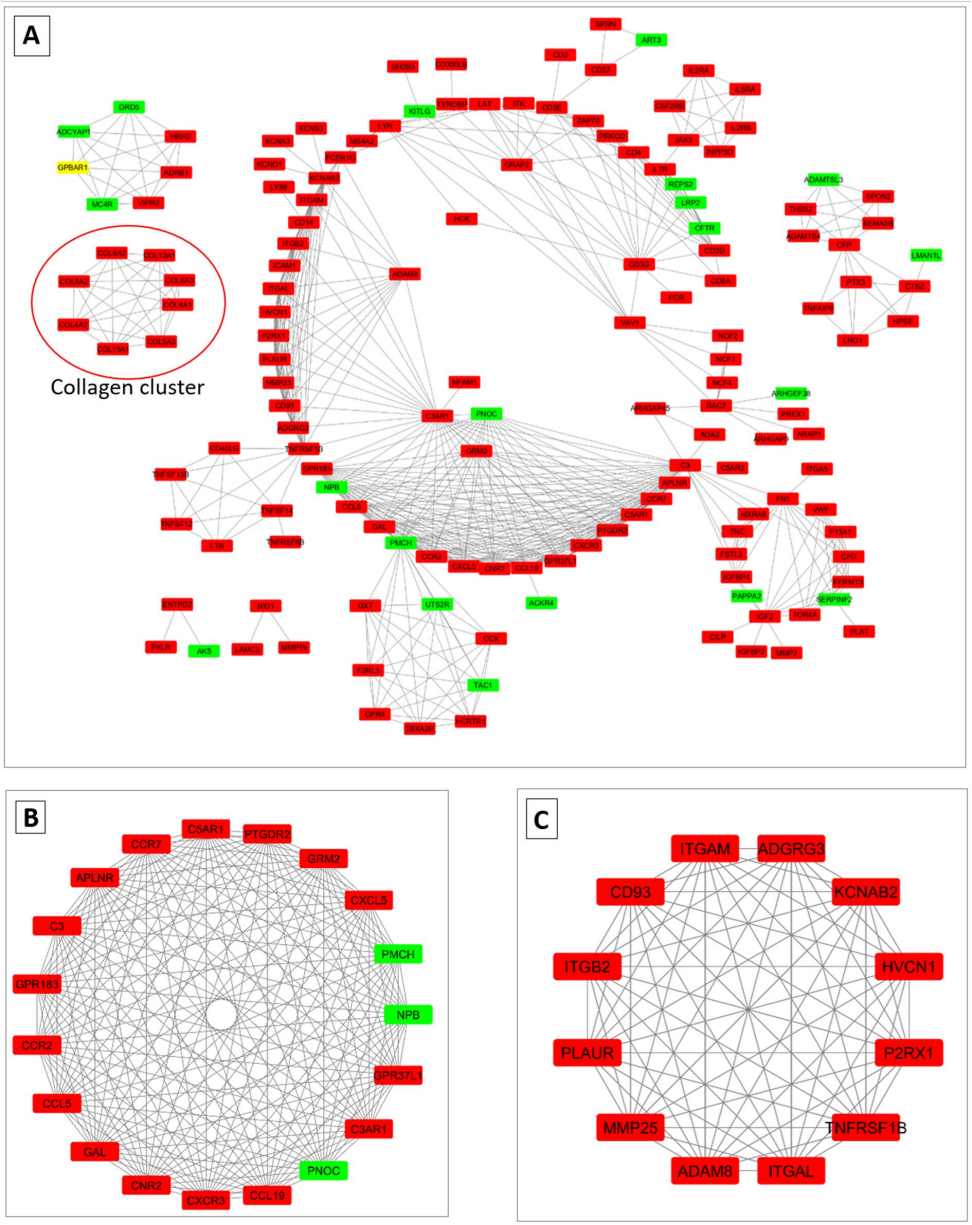
Protein–protein interaction (PPI) network and hub clustering modules. Red color represents genes up-regulated in anagen and green color represent genes downregulated in the anagen (**A**) The PPI network of overlapping differentially expressed genes between telogen and anagen. (**B**) Cluster 1 (MCODE score = 19). (**C**) Cluster 2 (MCODE score = 12).

To identify the possible protein–protein interaction (PPI) between DEGs, STRINGDB^24^ was utilized. The PPI network of DEGs consisted of 149 genes and 563 interactions (Fig. 2A). Two topological features Maximal Clique Centrality (MCC) and degree were calculated to identify key nodes. Higher the two quantitative values of a gene, the more important it is in the PPI network. The top nodes ranked by degree and MCC were identified, which included C–C Motif Chemokine Receptor 7 (*CCR7*), C-X-C Motif Chemokine Ligand 5 (*CXCL5*), Complement C3(*C3*), C–C Motif Chemokine Ligand 19(*CCL19*), Complement C3a Receptor 1 (*C3AR1*), Complement C5a Receptor 1 (*C5AR1*), C–X–C Motif Chemokine Receptor 3 (*CXCR3*), Pro-Melanin Concentrating Hormone (*PMCH*), G Protein-Coupled Receptor 183 (*GPR183*), G Protein-Coupled Receptor 183 (*GPR37L1*). The enrichment analysis of key genes in the PPI network suggests their role in *Chemokine signaling pathway (FDR* = 0.00001) and *Cytokine-cytokine receptor interaction (FDR* = 0.0007). Chemokine pathway might be associated with a unique immune milieu responsible for hair follicle immune privilege that occurs in the anagen phase to spare the cell proliferation in the follicle from the possible immune reaction.

Additionally, a cluster of 8 closely related collagen genes were detected which were all up-regulated in anagen, suggesting that collagen genes may play an important role in hair cycle and maintaining continuous hair growth (Fig. 2A—solid red outlined). In order to identify other significant clusters from the PPI network a module analysis was performed and the top 2 modules with high scores were selected. Cluster 1 contained 19 nodes and 171 interactions (Fig. 2B). KEGG enrichment analysis suggest, genes in cluster 1 were enriched in ‘*Neuroactive ligand-receptor interaction *(*FDR* = 9.8 × 10–8)’, ‘*Chemokine signaling pathway *(*FDR* = 3.5 × 10–5)’ and ‘*Cytokine-cytokine receptor interaction *(*FDR* = 4.5 × 10–4)’. GO enrichment analysis suggest genes in cluster 1 was closely related to ‘*chemokine-mediated signaling *(*FDR* = 5.1 × 10–6)’, ‘*immune response *(*FDR* = 0.00014)’, ‘*regulation of cell migration *(*FDR* = 0.0003)’ and ‘*nervous system development *(*FDR* = 0.0181)’. Nervous system and hair follicle epithelium share a common ectodermal origin; therefore, it is reasonable to ask whether neurohormones are also involved in hair growth control. Cluster 2 contained 12 genes and 66 interactions (Fig. 2C), the genes were mainly implicated in ‘*RAP1 signaling pathway *(*FDR* = 0.0011), ‘*Cell adhesion molecules *(*FDR* = 0.0006)’and ‘*integrin- mediated signaling *(FDR = 0.01)’. Integrins are adhesion receptors allow cells to sense and respond to microenvironmental signals which could be essential in telogen to anagen transition in Pashmina goats as well.

**Figure 3:**
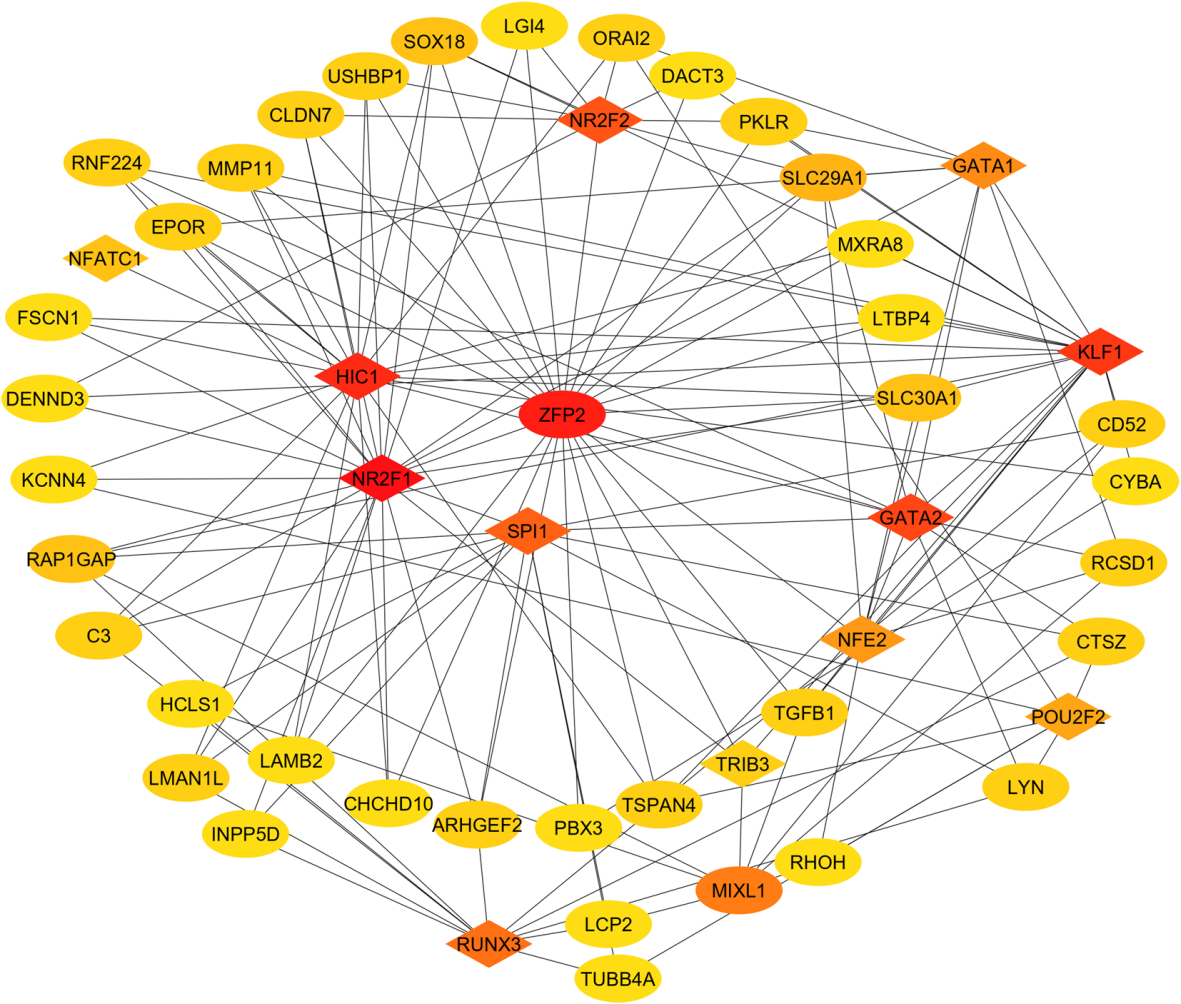
TF-gene regulatory network (developed using ref.^19,20^). The eclipse in the TF-gene network represented mRNA and the diamonds represented TFs. Color represents degree (red to yellow represent decreasing order of degree).

The hair cycle is a highly regulated process, earlier studies suggest growth factors, cytokines, hormones, and transcription factors (TFs) play a critical role in mediating overall hair cycle^3^. In this study, a TF-regulatory network was generated (Fig. 3) to identify the critical TFs mediating anagen induction in Pashmina goats^25–27^. The |(log (FC))| and degree were used to identify key TFs in regulating hair cycle. The higher the two quantitative values of a gene, the more important it is in the TF-regulatory network. The TF-gene interaction network consisted of 50 nodes and 150 interactions (Fig. 3). The top-ranked TFs were Nuclear Receptor Subfamily 2 Group F Member 1 (*NR2F1*), Zinc Finger Protein (*ZFP2*), HIC ZBTB Transcriptional Repressor 1 (*HIC1*), Kruppel Like Factor 1 (*KLF1*), GATA Binding Protein 2 (*GATA2*), Nuclear Receptor Subfamily 2 Group F Member 2 (*NR2F2*), Spi-1 Proto-Oncogene (SPI1), RUNX Family Transcription Factor 3 (*RUNX3*), Mix Paired-Like Homeobox (*MIXL1*) and GATA Binding Protein 1 (*GATA1*). Interestingly, ZFP2 was the only gene down-regulated in the active growth phase.

### Validation of DEGs with qPCR

The differential expression of 5 genes namely; *HAS1, TRIB2, P2RX1, PRG4, CNR2, MMP25, GIPC2, CDO1,* and *MC4R* was validated by qRT-PCR. Primer pairs for these genes are listed in Supplementary Table 4. The expression profile of these genes obtained by qRT-PCR showed a similar trend (Pearson’s correlation coefficient = 0.88) with the RNA-seq results, thereby validating the RNA-seq results (Supplementary Fig. 2).

## Discussion and conclusion

Hair growth has been reported to be regulated by a complex mechanism involving multiple endogenous and exogenous factors. Understanding the genetic basis of fibre production phenotypes could contribute to the improvement of the fibre production efficiency in Pashmina goats and also help in identifying molecular determinants involved in anagen initiation. In this study, skin samples specifically containing SHFs were collected during anagen and telogen to identify key regulators and pathways associated with the telogen–anagen (TA) transition.

Enrichment analysis of the DEGs for the TA transition revealed that the transition involves as many as 22 unique pathways. Among the enriched pathways Wnt, NF-B, JAK-STAT, MAPK and Calcium signaling are known in regulating hair follicle morphogenesis and development. Wnt acts as master regulating during hair follicle morphogenesis. NF-κB and Wnt signaling pathways play a vital role in hair follicle initiation and development. Strong NF-κB activity was detected in the secondary hair germ of late telogen and early anagen HFs in mouse^28^, suggesting a potential role for NF-κB in HF stem/progenitor cell activation during anagen induction. The present study also suggests a possible role of NF-κB in anagen induction. In the present study, the up-regulation of *IL36RN* during telogen which inhibits NF-κB suggests its potential role in maintaining telogen in Pashmina goats. The JAK-STAT pathway has recently been reported to be important in anagen induction and is responsible for jump-starting the hair cycle^29,30^. Up-regulation of *IGF2* and enrichment of MAPK in anagen suggests the role of *IGF2* in promoting anagen via MAPK signaling. Calcium signaling pathway was also significantly enriched. A transient role of Ca is reported during HF cycling^31^. Also, the melanogenesis pathway was significantly enriched in TA transition suggesting that melanogenesis pathway also resonate with hair cycle in Pashmina goats^32^.

The most distinguishing feature of the present study was significant enrichment of ECM-receptor interaction and Cell adhesion molecules (CAMs). Hair follicle growth depends on the interaction between CAMs and ECM-receptors (enriched by 18 genes and 24 genes respectively), it was found that *ITGAV* was strongly up-regulated in telogen samples, suggesting the potential role of *ITGAV* in maintaining hair follicle in the telogen phase. Protein–protein interaction network (Fig. 2A) suggests the potential role of Integrin molecules like *ITGB7, ITGA5, ITGA9* and *ITGA11* in anagen-induction. In human adipose stem cells, the increase in *ITGAV* causes reduced cell proliferation^33^. However, the increased levels of *ITGA5* activates cell proliferation and differentiation. Our data might postulate a functional role of *ITGAV* and *ITGA5* in hair cycle.

Moreover, several other pathways mediating cellular adhesion, cell differentiation and proliferation were significantly enriched in Pashmina anagen induction, including ‘pathways regulating pluripotency of stem cells’, ‘PI3K-Akt’, ‘Hippo’, ‘RAP1’, ‘RAS’, ‘cGMP-PKG’, ‘cAMP’, ‘Neuroactive ligand-receptor interaction’ and ‘Cytokine-cytokine receptor interaction’. PI3K-Akt signaling promotes cell proliferation in animal tissues by mediating Hippo signaling^34^, up-regulating of genes positively mediating PI3K-Akt and downregulation of genes regulating Hippo signaling during anagen is therefore plausible. In our data, IGF1R was identified to be up-regulated during anagen which acts as a key regulator for PI3K-Akt, MAPK, RAS, RAF signaling pathways suggesting its importance in TA transition^35^.

A total of 10 key TFs were identified from the TF-regulatory network namely, *GATA2, GATA1, RUNX3, NR2F1, NR2F2, HIC1, SPI1, MIXL1, ZFP2* and *KLF1*. Interestingly, *ZFP2* was the only gene downregulated in the active growth phase (anagen). The GATA family of TFs plays an important role during embryonic development, including cell fate decision and tissue morphogenesis. *GATA1* knockdown mouse exhibits developmental arrest and cell death^36,37^, network analysis suggest GATA1 interact with *GATA2* may be for controlled cell differentiation and proliferation. *RUNX3* plays a critical role in normal hair growth, a *RUNX3* knockdown mice shows significant change in hair structure and composition^38^. This study also suggests the potential role of more diverse TFs (like *NR2F1, NR2F2, HIC1, SPI1, MIXL1, ZFP2* and *KLF1*) in promoting anagen.

## Supplementary Information

Supplementary Information 1.

Supplementary Table 2.

Supplementary Table 3.

## Data Availability

The sequencing data is available in NCBI under accession number GSE164100.
