# Supplementary material for: Identification of potential key genes and pathways associated with the Pashmina fiber initiation using RNA-Seq and integrated bioinformatics analysis
Source: Sci Rep. 2021 Jan 19;11:1766. doi: 10.1038/s41598-021-81471-6 (PMC7815713; doi:10.1038/s41598-021-81471-6)
Supplement: Supplementary file 1 — Supplementary Information 1. [file 41598_2021_81471_MOESM1_ESM.docx]

**Identification of potential key genes and pathways associated with the Pashmina fiber initiation using RNA-Seq and integrated bioinformatics analysis**

# Basharat Bhat1,2,*, Mifftha Yaseen3, Ashutosh Singh2, Syed Mudasir Ahmad1, and Nazir A. Ganai1,*

1Division of Animal Biotechnology, Sher-e-Kashmir University of Agricultural Sciences and Technology of Kashmir, FV.Sc and A.H, Shuhama, Jammu and Kashmir, 190016, India

2Department of Life Science, Shiv Nadar University, Gautam Buddha Nagar, UP, 201314, India

3Department of interdisciplinary sciences, Jamia Hamdard University, Delhi, 110062, India

*[bb284@snu.edu.in](mailto:bb284@snu.edu.in)

*[naganai@skuastkashmir.ac.in](mailto:naganai@skuastkashmir.ac.in)

**Table 1.** Quality control and alignment statistics of RNA-seq reads to *Capra hircus* reference genome

| S. No | Sample ID | Read orientation | Number of Reads  (in millions) | Cleaned Paired Reads  (in millions) | Total mapped |
| --- | --- | --- | --- | --- | --- |
| 1. | Anagen_PG1 | **R1** | 49.51 | 45.08 | 97.15% |
|  |  | **R2** | 49.51 |  |  |
| 2. | Anagen_PG2 | **R1** | 51.13 | 48.01 | 97.64% |
|  |  | **R2** | 51.13 |  |  |
| 3. | Anagen_PG3 | **R1** | 49.81 | 47.21 | 97.63% |
|  |  | **R2** | 49.81 |  |  |
| 4. | Anagen_PG4 | **R1** | 52.73 | 48.73 | 97.56% |
|  |  | **R2** | 52.73 |  |  |
| 5. | Anagen_PG5 | **R1** | 45.91 | 43.28 | 97.71% |
|  |  | **R2** | 45.91 |  |  |
| 6. | Anagen_PG6 | **R1** | 36.18 | 33.18 | 96.32% |
|  |  | **R2** | 36.18 |  |  |
| 7. | Anagen_PG7 | **R1** | 52.46 | 50.59 | 97.65% |
|  |  | **R2** | 52.46 |  |  |
| 8. | Anagen_PG8 | **R1** | 59.71 | 54.10 | 97.72% |
|  |  | **R2** | 59.71 |  |  |
| 9. | Anagen_PG9 | **R1** | 43.58 | 34.25 | 98.85% |
|  |  | **R2** | 43.58 |  |  |
| 10. | Anagen_PG10 | **R1** | 44.59 | 41.71 | 98.21% |
|  |  | **R2** | 44.59 |  |  |
| 11. | Telogen_PG1 | **R1** | 47.79 | 42.74 | 98.60% |
|  |  | **R2** | 47.79 |  |  |
| 12. | Telogen_PG2 | **R1** | 56.14 | 51.92 | 97.49% |
|  |  | **R2** | 56.14 |  |  |
| 13. | Telogen_PG3 | **R1** | 49.32 | 46.66 | 98.19% |
|  |  | **R2** | 49.32 |  |  |
| 14. | Telogen_PG4 | **R1** | 63.38 | 59.58 | 98.01% |
|  |  | **R2** | 63.38 |  |  |
| 15. | Telogen_PG5 | **R1** | 57.28 | 54.25 | 97.79% |
|  |  | **R2** | 57.28 |  |  |
| 16. | Telogen_PG6 | **R1** | 48.38 | 45.44 | 97.73% |
|  |  | **R2** | 48.38 |  |  |
| 17. | Telogen_PG7 | **R1** | 59.28 | 55.89 | 97.93% |
|  |  | **R2** | 59.28 |  |  |
| 18. | Telogen_PG8 | **R1** | 64.33 | 60.63 | 97.50% |
|  |  | **R2** | 64.33 |  |  |
| 19. | Telogen_PG9 | **R1** | 72.28 | 68.80 | 97.68% |
|  |  | **R2** | 72.28 |  |  |

| 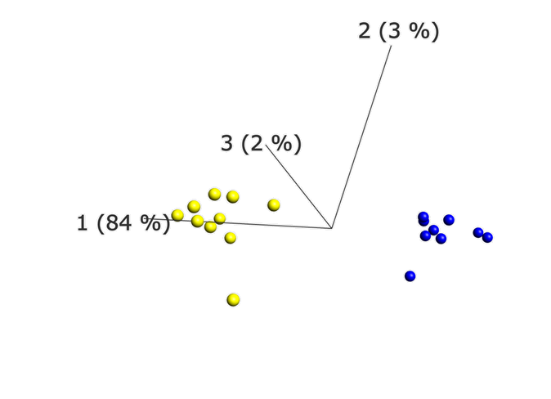 | 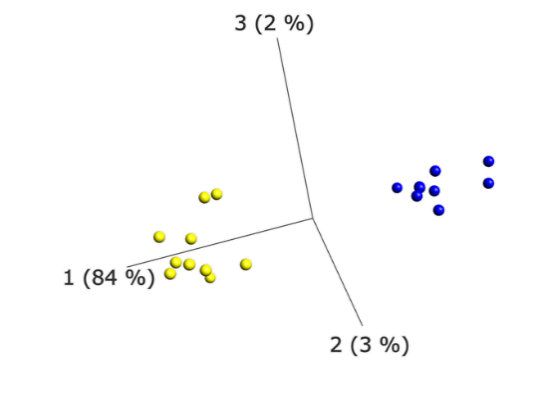 | 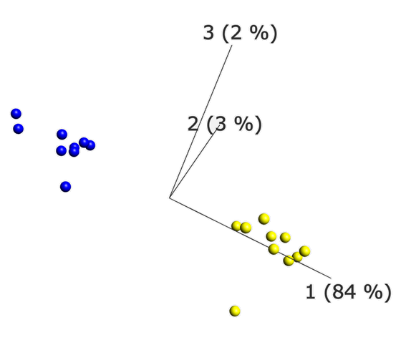 |
| --- | --- | --- |
| PCA 1 | PCA 2 | PCA 3 |

**Figure 1.** PCA plots of RNA-sequencing data showing the characteristics of samples according to gene expression (TMM normalized) levels.

**Table 4.** List of primers used in qPCR validation

| **Gene name** | **Primer Sequence** | **Size** |
| --- | --- | --- |
| GAPDH | F 5' GGCGTGAACCACGAGAAGTA 3'  R 5' GGCGTGGACAGTGGTCATAA 3' | 141 bp |
| HAS1 | F 5' GGGCTATGCCACCAAGTACA 3'  R 5' CAGAGCGCATTGTAGAGCCA 3' | 132 bp |
| TRIB2 | F 5' TCCGTACCTGCAAGAAGCTG 3'  R 5' GACCCGAGTCCTTTCTTCGT 3' | 152 bp |
| P2RX1 | F 5' AAGACCTTGCACCCTCTGTG 3'  R 5' AGTGCCAGTCGATGGTGATG 3' | 121 bp |
| PRG4 | F 5' CGCTGCTTCGAATCCTTTGC 3'  R 5' GGTGAACGTTTGGTTGCTGA 3' | 194 bp |
| CNR2 | F 5' CCTCATCGTGTCCTCACACC 3'  R 5' GCTTTGGAATCCACGCCATC 3' | 144 bp |
| MMP25 | F 5' GCAACACGGGTGACACCTA 3'  R 5' AGACTTCCCACAGCCACAAC 3' | 351 bp |
| MC4R | F 5' ATTCCGTCATCGACCCTCTG 3'  R 5' GCACTGCAGTTTGTCCCCAT 3' | 139 bp |
| GIPC2 | F 5' GAGATCGAAAGGTCCTGCCA 3'  R 5' CTGGGAACGCAAAGTCTCCA 3' | 209 bp |
| CDO1 | F 5' GAGGGAAAACCAGTGTGCCT 3'  R 5' GATCAAAGGCGTGGCATGTG 3' | 131 bp |

**
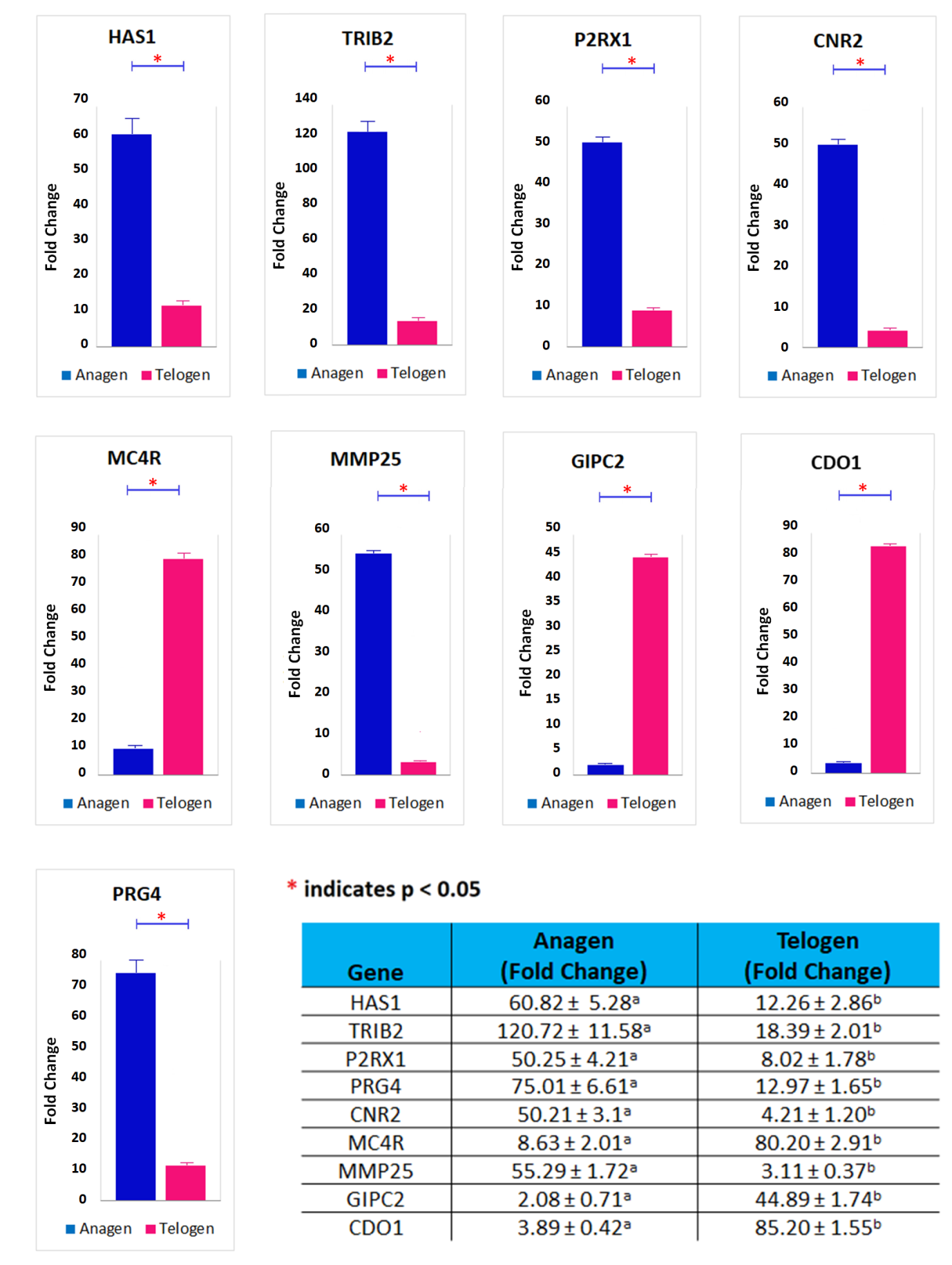
**

**Figure 2.** Comparison of the expression levels of DEGs during anagen and telogen obtained by qPCR detection method.
